# Effectiveness of Peer Education of Tooth-Brushing in Children

**Published:** 2011-12-01

**Authors:** F Ghaderi, M Oshagh, P Ashtiju, A Bagherpur

**Affiliations:** 1 Department of Pedodontics, Shiraz University of Medical Sciences, Shiraz, Iran; 2 Department of Orthodontics, Orthodontic Research Center, Dental School, Shiraz University of Medical Sciences, Shiraz, Iran; 3 Dentist, Shiraz University of Medical Sciences, Shiraz, Iran

**Keywords:** Peer education, Tooth-Brushing, Children

Dear Editor,

Dental caries and periodontal diseases are still considered the most frequent oral ailments and considered as public health problems, which could result in the loss of dental structures and its support. Although dentistry has experienced great advances, the prevention of dental caries and periodontal diseases are still the best form of treatment.[1] Therefore, education of patients deserves the most attention in preventive dentistry. As children and teenagers are more receptive to new knowledge, they can respond to trainings with greater efficiency. However, when working with the children, the education methods must be attractive and arouse the children's attention.[1]

The tendency for people to pay attention to their peers also may be utilized in educating for oral health.[2] Since the 1960s, peer education became an alternative approach to the expert status of the professional[3][4][5] and is originated from a fact that young people learn a lot from one another as part of their everyday lives.[3][5][6] In Iran, 52% of the population are younger than 20 years. So Iran is one of the youngest populations in the world[7] which the level of oral health is still not satisfactory, particularly among children.[8]

In this cross-sectional study, 117 school children of both genders (59 girls and 58 boys), aged 8 years were selected randomly by random cluster sampling method from primary schools of four areas in Shiraz, Fars Province, Southern Iran. One school was selected from each area and about 30 children were selected from each school. Only children whose parents had signed the consent form were included in this study. The selected students were randomly divided to two groups including 57 children: Dentist education and 60 children: Peer education.

First, in group 1; the students were educated by one general dentist about tooth brushing. Then from the students of that group, one volunteer child with good attitudes and communication ability was chosen to educate tooth brushing to the second group. In both groups, after education; comparable toothbrushes with toothpaste were delivered to accomplish tooth brushing which they educated. The plaque index was scored according to manufacturer's instructions before and immediately after the tooth brushing.[1][9] To evaluate the effect of education method on dental plaque index decrease, Student's t test was used. The significance level was 0.05.

There was no significant difference between the mean score of plaque index before education in both groups (p=0.578). Also, the mean score of plaque index after education in both groups was not significantly different (p=0.674). In both groups, the mean scores of plaque index reduced and the differences of plaque index after and before education were not significantly different in both groups (p=0.374).

The reduction of dental plaque index in both groups of this study is in agreement with Rodrigues et al.'s findings.[1] Also Ehudin et al. concluded that peer- teaching program can be an effective mode of instruction.[10] Reinhardt et al. found that after peer teaching, there was a significant increase concerning tooth-brushing time, performance of circular tooth brushing movements, and systematic cleaning of all dental surfaces.[11] In another study, Reinhardt et al. stated that tutoring peers can function as a form of empowerment and can establish a strong sustained health engagement.[12]

The difference in final mean scores for plaque index can be related merely to the difference in the initial mean scores of groups, something which resulted from the sampling[2] but in our study, the mean score of plaque index in both group was not significantly different before education. The oral hygiene education should be given as a separate part of the treatments and also education should not be accomplished at random. To obtain the expected results, the dentist must select the appropriate methods compatible with the target public.[1] In a large education program, it would be impossible to instruct all participants individually. The use of methods such as peer-education could enable one to provide the program effectively for a wide audience.[2] To evaluate the effects of peers in children, Dorri et al. concluded that adolescents who had stronger ties with their friends were more likely to brush their teeth twice or more a day.[13] Also, during education of children, it should be attractive which arousing their attention and it must be reminded that the direct instruction used in isolation was not as effective on dental plaque index reduction as when associated to indirect instruction.[1] In Rodrigues et al. study, all the motivation methods (included smiling robot, slides and macromodels) promoted significant decrease of plaque index in children and among these methods, the robot was the one that provided the best results.[1]

Since there was no significant difference in effectiveness between peer and dentist education, this method of education can be recommended for education of oral hygiene care in general population.
